# Editorial: Strategies to improve management of ventricular tachycardias

**DOI:** 10.3389/fcvm.2023.1279076

**Published:** 2023-09-05

**Authors:** Alexander H. Maass, Yuri Blaauw, Bart A. Mulder, Katja Zeppenfeld

**Affiliations:** ^1^ From the Department of Cardiology, University of Groningen, University Medical Center Groningen, Groningen, Netherlands; ^2^ Department of Cardiology, Leiden University Medical Center, Leiden, Netherlands

**Keywords:** risk, substrate, imaging, catheter ablation, magnetic resonance imaging

**Editorial on the Research Topic** Strategies to improve management of ventricular tachycardias

Ventricular tachyarrhythmias are the main cause of sudden cardiac death and can occur in the presence of acute myocardial ischemia or chronic electrical or structural arrhythmic substrates. In the last decade advances in risk stratification and treatment of ventricular arrhythmias have been published and recent guidelines have summarized the state of evidence (1). There are, however, many gaps in the evidence; some of them are addressed in this special issue. The topics can be divided into two main sections: improvements in (1) risk stratification and (2) treatment of ventricular tachycardia (VT).

It is of utmost importance to identify patients at risk of developing VT/sudden cardiac death and offer an implantable cardioverter-defibrillator (ICD) in these high-risk patients, who have an otherwise good life expectancy. In ICD carriers recurrent VT is a major concern. Even hemodynamically tolerated VT, can lead to life-threatening situations or acute heart failure, if untreated. In addition, despite a preserved blood pressure, VT can be severely symptomatic and cause dangerous situations e.g., if the patient is driving or in an otherwise exposed position. ICD shocks impact quality of life and are a main cause of anxiety and depression in ICD carriers. Thus, also in primary prevention ICD patients early catheter ablation of VT may be beneficial before the first episode of sustained VT even though it is currently mainly performed in patients with recurrent VTs (2). Risk scores for prediction of VT/SCD have been developed for several specific conditions such as hypertrophic cardiomyopathy, arrhythmogenic cardiomyopathy, and other genetic cardiomyopathies and their use is recommended in the guidelines, if validated (1). The largest patient group at risk of developing VT, however, are patients with prior myocardial infarction. Trying to develop a simple risk score has proven difficult and left ventricular ejection fraction (LVEF) remains the best (but imperfect) predictor of sudden cardiac death (3). Many patients with an LVEF of less than 35% will never experience life-threatening arrhythmias whereas myocardial infarct scars can be severely arrhythmogenic in the presence of preserved or mildly reduced LVEF. Non-invasive studies combined with invasive programmed stimulation may help to identify patients at high risk in this specific group of patients (4). Advanced imaging techniques, in particular cardiac magnetic resonance imaging (CMR) might improve risk stratification in patients with an ischemic arrhythmic substrate. Noordman et al. could demonstrate that myocardial scar characterization by CMR including total and borderzone scar mass predicts ICD therapy in patients with ischemic heart failure (Noordman et al.). Moreover CMR scar characterization might identify patients at risk that are currently not considered candidates for prophylactic ICD because of LVEF >35% (Roca-Luque & Mont-Girbau). In addition to CMR based risk stratification, this imaging technique is also evolving as an intra-procedural imaging technique during catheter ablation (Roca-Luque & Mont-Girbau).

Patients that have survived out-of-hospital cardiac arrest due to documented ventricular fibrillation not related to acute ST-elevation myocardial infarction are usually implanted with an ICD. Risk stratification in these patients is still important as many patients who presented with ventricular fibrillation may have initially monomorphic VT, degenerating into VF and are potential candidates for early VT ablation. Non-invasive programmed stimulation to induce monomorphic VT via the ICD might be an attractive diagnostic tool to identify patients at high risk for future events (5) and even offer early treatment with antiarrhythmic drugs or VT ablation. Lastly, even among patients with current secondary prevention ICD indication, many will never develop recurrent arrhythmias and identification of a low-risk subgroup might prevent unnecessary ICD implantations with the inherent reduction in patients' quality of life and increase in health care expenditures (6). Also in this patient group, CMR may aid in identifying patients at low risk of recurrence (7).

Preventing VT recurrence Treatment of VT has proven difficult because of the limited efficacy of antiarrhythmic medication. Although acute episodes of VT can be treated with class I or III antiarrhythmic drugs or amiodarone, their value in preventing VT is limited due to a lack of efficacy and/or undesired side effects. Catheter ablation has developed as an important treatment modality to prevent recurrence of VT. 3D mapping systems and multielectrode catheters allow for rapid substrate and activation mapping for hemodynamically tolerated VTs. Technical advances have resulted in improved safety and efficacy of ablation. Functional substrate mapping strategies can improve substrate delineation in particular in patients with post-infarct scars. In general, the majority of available data are derived from patients with prior myocardial infarction and cannot be easily extrapolated to VT substrates in patients with different non-ischemic aetiologies. In particular patients with DCM or HCM have a less favorable outcome. For these patients pre- and intraprocedural imaging can be beneficial. The type of anesthesia, intraprocedural mapping and the use of imaging, as well as the type of catheters used differ between centers. After more than 3 decades, VT ablation is still not a standardized procedure and there are no studies comparing available mapping strategies. A systematic approach of mapping and ablation, tailored to the specific underlying substrate is important and randomized studies comparing currently applied and promoted mapping and ablation strategies desirable (Subramanian et al.).

To identify the underlying electrical and molecular mechanisms that predispose patients to life-threatening arrhythmias is a crucial step to develop novel antiarrhythmic drugs. Heterogeneity of ion channel or gap junction proteins can contribute to changes in cardiac conduction and repolarization. Specific animal models can aid in identifying these mechanisms (Boulaksil et al.).

In summary, techniques for identifying patients at risk of developing VT as well as standardized catheter ablation techniques are developing. Electrical and molecular mechanisms of VT are being investigated and this might lead to the design of novel antiarrhythmic drugs. However, many gaps in the evidence remain and we encourage investigators in the field to continue their efforts on mechanistic studies as well as to design prospective trials on unanswered questions.
